# Evaluation of the Brain Function State During Mild Cognitive Impairment Based on Weighted Multiple Multiscale Entropy

**DOI:** 10.3389/fnagi.2021.625081

**Published:** 2021-07-30

**Authors:** Rui Su, Xin Li, Yi Liu, Wei Cui, Ping Xie, Ying Han

**Affiliations:** ^1^Key Laboratory of Measurement Technology and Instrumentation of Hebei Province, Institute of Electric Engineering, Yanshan University, Qinhuangdao, China; ^2^Handan First Central Hospital, Handan, China; ^3^Department of Neurology, Xuanwu Hospital of Capital Medical University, Beijing, China

**Keywords:** mild cognitive impairment, electroencephalogram, multiscale entropy, weighted multiple multiscale entropy, neurofeedback training

## Abstract

The mild cognitive impairment (MCI) stage plays an essential role in preventing the progression of older adults to Alzheimer's disease. In this study, neurofeedback training (NFT) is applied to improve MCI brain cognitive function. To assess the improvement effect, a novel algorithm called Weighted Multiple Multiscale Entropy (WMMSE) is proposed to extract and analyze the electroencephalogram (EEG) features of patients with MCI. To overcome the information loss problem of traditional multiscale entropy (MSE), WMMSE fully considered the correlation of the sequence and the contribution of each sequence to the total entropy. The experimental group composed of 39 patients with MCI was subjected to NFT for 10 days during two sessions. The control group included 21 patients with MCI without any intervention. The Lempel-Ziv complexity (LZC) was used for primary assessment, and WMMSE was used to accurately analyze the effect of NFT. The results show that the WMMSE values of F4, C3, C4, O1, and T5 channels post-NFT are higher compared with pre-NFT and significant differences (*P* < 0.05). Moreover, the cognitive subscale of the Montreal Cognitive Assessment (MoCA) results shows that the post-NFT score is higher than the pre-NFT in the vast majority of the patients with MCI and significant differences (*P* < 0.05). When compared with the control group, the WMMSE values of the experimental group increased in each channel. Therefore, the NFT intervention method contributes to brain cognitive functional recovery, and WMMSE can be used as a biomarker to evaluate the state of MCI brain cognitive function.

## Introduction

Mild cognitive impairment (MCI) is an early stage of Alzheimer's disease (AD), and it is a critical target for preventing the progression to AD in older adults (Cheng et al., 2015; Li Y. et al., 2019). Early interventions can reduce the risk of progression to AD, such as medication, physical rehabilitation, behavior, and cognitive therapy (Tsolaki et al., 2009; Fessel, 2019; Delmastro et al., 2020). In recent studies, increasing attention has been paid to neurofeedback training (NFT), which has improved brain dysfunction and clinical symptoms (Wang and Hsieh, 2013).

Neurofeedback training converts the electroencephalogram (EEG) data into visual and auditory signals, and participants selectively enhance or inhibit EEG frequency during training to achieve brain regulation (Monderer et al., 2002). NFT has been successfully applied to brain function–related diseases such as attention deficit hyperactivity disorder (ADHD) (Deiber et al., 2020; Janssen et al., 2020), autistic spectrum disorder (ASD) (Kang et al., 2020), and epilepsy (Ouyang et al., 2020). However, few studies have applied NFT in MCI rehabilitation. Patients with AD receiving NFT possess stable cognitive function and can enhance their information recognition and memory (Luijmes et al., 2016). A shooting game of the NFT system has been developed. The peak frequency of the alpha band of all subjects increased significantly after NFT, indicating that NFT could improve attention and cognitive function (Liu et al., 2014). NFT, as a training technique, succeeded in decreasing the ratio of theta/alpha power of patients with MCI and improving cognitive functions. This study demonstrated that NFT could be used as a rehabilitation training method to improve the memory and attention of patients with MCI (Jirayucharoensak et al., 2014). However, the research group that performed the study did not focus on the method used to assess the effect of NFT.

Electroencephalogram is a non-invasive technique with a high temporal resolution, which can record the instantaneity and rapidity of brain signals (Maturana-Candelas et al., 2019). The complexity of EEG signals can reflect the changes of brain characteristics in different cognitive states. Entropy is an important characteristic parameter used to analyze the EEG complexity and can include Shannon's entropy, approximate entropy (ApEn) (Gao et al., 2020), and sample entropy (SampEn) (Xu et al., 2020). Multiscale entropy (MSE) quantifies the unpredictability of EEG signal by coarsening or averaging granulation and extracts more dimensional information from EEG signals (Costa and Goldberger, 2015). The EEG signals of AD are analyzed by extracting MSE and refined multiscale spectral entropy (rMSSE) features, and the results showed that the features contribute to tracking the progression of AD (Maturana-Candelas et al., 2019). However, there are some disadvantages to MSE, such as unstable trend and information loss. To solve this problem, we proposed a novel entropy algorithm called Weighted Multiple Multiscale Entropy (WMMSE), which can extract more detailed information by increasing the weight and multiple to overcome the information loss and optimize the NFT evaluation effect.

In this study, a new analysis method is proposed to evaluate the brain function of patients with MCI post-NFT. First, we adopted an NFT rehabilitation method based on EEG signals from patients with MCI. Second, the WMMSE was proposed as a biomarker for patients with MCI to evaluate the effect of NFT. The two main contributions of this study are as follows:

1. The NFT was designed based on EEG signals for improving the clinical symptoms of MCI. Meanwhile, the experimental group had performed NFT in two sessions.
2. We proposed a novel EEG feature extraction algorithm called WMMSE, which can mine more detailed information from the original EEG signals. Different from the existing studies, the WMMSE adds two parameters (i.e., weight and multiple). Therefore, the simulation results showed that WMMSE extracts more detailed information, and the effect of NFT was evaluated accurately.

## Materials and Methods

### Participants

Sixty right-handed patients with MCI (mean age, 65 ± 3 years; range, 60–70 years; 29 men) participated in this study, which consists of a control group (i.e., 21 subjects) and an experimental group (i.e., 39 subjects). The EEG signals of MCI were obtained from the First Hospital of Hebei Medical University. The neurosurgeon at the hospital diagnosed patients with MCI. These patients received the Cambridge Cognitive Examination (CAMCOG) and scored above 60. In addition, the inclusion criteria included the Mini-Mental State Examination (MMSE), Montreal Cognitive Assessment (MoCA) scores, and the Daily Living Test. The subjects underwent an MRI or CT examination to exclude focal lesions in the brain. They did not have a history of other neurological diseases (e.g., depression, epilepsy, and brain injuries). At the same time, patients with MCI did not take any nerve drugs. All participants provided written informed consent, and the whole NFT procedure was described before the experiment. The experiment was conducted in accordance with the Declaration of Helsinki and was approved by the ethical review board of Yanshan University.

### EEG Recording and Pre-processing

**Figure 1:**
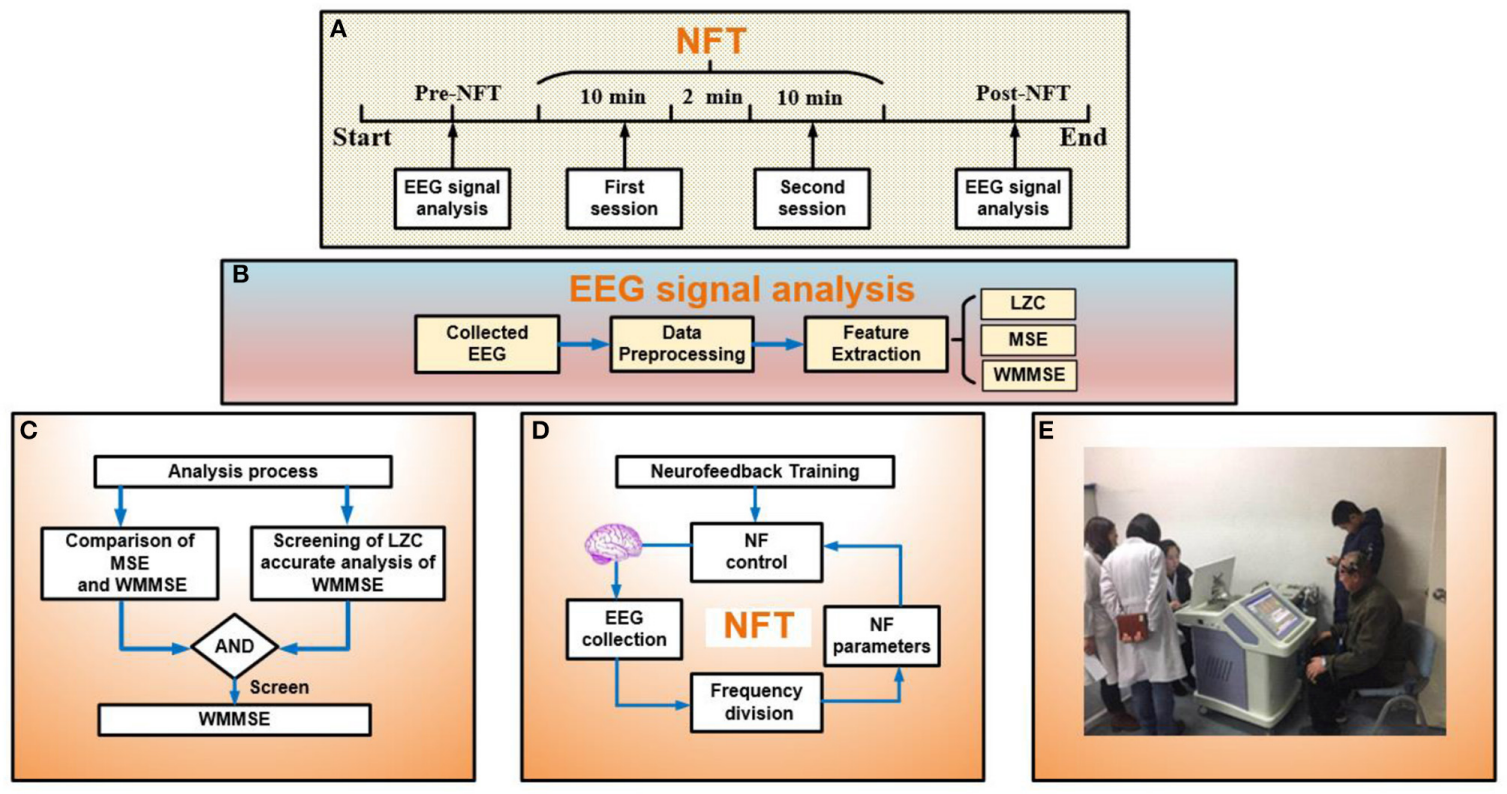
**(A)** Experimental process: analysis electroencephalogram (EEG) signal pre- and post- neurofeedback training (NFT) and the session of NFT. **(B)** EEG signal analysis: collected EEG, data preprocessing, and feature extraction. **(C)** Analysis process: evaluating the effect of NFT. Part 1 is the comparison between MSE and WMMSE. In part 2, to evaluate the effect of NFT, the first step is screening by LZC, and the second step is the accurate analysis of WMMSE. **(D)** The process of NFT. **(E)** Clinical application of NFT.

In Figure 1A, the NFT task includes two sessions of attention training with a 120 s break between two sessions, and each attention training session lasts for 10 min. Participants in the experimental group needed to complete the NFT task once per day for 10 days. Participants in the control group had no interventions performed on them. The process of EEG signal processing pre- and post- NFT is shown in Figures 1B,C. EEG signals were recorded in a quiet room. The subjects were awake, seated on a comfortable chair, and relaxed in an eyes-open state without other activities such as shaking the head, gritting teeth, and facial movements.

The NFT process is shown in Figure 1D. The NFT of MCI patients included two sessions: relaxation training and attention training. The NFT device adopted the Alzheimer's disease training system (ADTS) jointly developed by the research group and Qinhuangdao Huisi Anpu Medical System (Figure 1E). A 16-channel digital EEG device (NT9200, XinTuo Company, Beijing) was used to collect EEG signals. The sampling rate was 1,000 Hz. The electrodes of 16 channels adopted the 10/20 international standards system and were distributed in the frontal, parietal, temporal, and occipital lobe (Ramirez and Vamvakousis, 2012). The 16 channels were FP1, FP2, F3, and F4 in the frontal lobe, F7, T3, and T5 in the left temporal lobe, F8, T4, and T6 in the right temporal lobe, C3, C4, P3, and P4 in the parietal lobe, and O1, and O2 in the occipital lobe.

EEG signals were processed in the MATLAB environment. The signals were filtered using the FieldTrip toolbox with a band frequency of 0.5–40 Hz. Different kinds of noise such as the power frequency, electro-oculogram (EOG) (Geetha and Geethalakshmi, 2012), electro-myogram (EMG) (Mortezaee et al., 2019), electro-cardiogram (ECG) (Nougarou et al., 2018), and galvanic skin response (GSR) (Sun et al., 2019) were removed in EEG signals. The statistical computations were performed using statistical product and service solutions (SPSS).

### Weighted Multiple Multiscale Entropy

Multiscale is the process of coarsely granulating the original signal to obtain information about different time scales. Compared with single-scale EEG signals, multiscale EEG signals can be mined to obtain more comprehensive signal information. However, the loss of original information cannot be prevented since the sequence length is considerably shortened during coarse granulation. By including the weight and multiple parameters, the information loss problem inherent to MSE is overcome, and more information on original signals can be mined. Therefore, the EEG signals are analyzed more accurately. A detailed description of the WMMSE method is provided below.

First, the algorithm constructs multiple sequences in each scale based on the moving average (MA). Second, the correlation coefficient of each sequence is calculated for each scale. Finally, the weight obtained by taking the correlation coefficient is calculated.

The signal sequences {*x*(1), *x*(2)⋯*x*(*i*)⋯*x*(*N*)} were defined with the time scale *s*, and the new signal sequence {*y*^(*s*)^} was obtained in each scale as follows:

(1) $$\begin{array}{r} \;y_{j}^{\left(s\right)}=\frac{1}{s}\overset{j+s-1}{\underset{i=j}{\sum }}x\left(i\right),\;1\leq j\leq N-s+1 \end{array}$$

Then, {*y*^(*s*)^} was made to construct multiple new sequences {*u*^(*s*)^} and length *l*. {*u*^(*s*)^} can be calculated as follows:

(2) $$\begin{array}{r} \begin{array}{c} u^{\left(s\right)}\left(i\right)=\left[u\left(i\right),\;u\left(i+s\times 1\right),\;u\left(i+s\times 2\right)......\;u\left(i+s\times \left(l-1\right)\right)\right]\\ \left(i=1,2,3......s\right),\;l=\left[\frac{N-s+1}{s}\right] \end{array} \end{array}$$

The signal sequences were defined with the time scale *s*, and the new signal sequence {*z*^(*s*)^} was obtained in each scale and length 1/*s*.

The sequences {*x*(1), *x*(2)⋯*x*(*i*)⋯*x*(*N*)} can be rewritten as follows:

(3) $$\begin{array}{r} z_{j}^{\left(s\right)}=\frac{1}{s}\overset{js}{\underset{i=\left(j-1\right)s+1}{\sum }}x\left(i\right),\;1\leq j\leq N/s \end{array}$$

Moreover, {*z*^(*s*)^} as a basic point, the correlation coefficient *R* of each series under each scale (Zhao and Xu, 2018) is defined as follows:

(4) $$\begin{array}{r} R\left(j\right)=\frac{\overset{n}{\underset{i=1}{\sum }}\left(z\left(i\right)-\overset{-}{z}\right)\left(u\left(i\right)-\overset{-}{u}\right)}{\sqrt{\overset{n}{\underset{i=1}{\sum }}{\left(z\left(i\right)-\overset{-}{z}\right)}^{2}*\overset{n}{\underset{i=1}{\sum }}{\left(u\left(i\right)-\overset{-}{u}\right)}^{2}}},\;1\leq j\leq s,\;n=N/s \end{array}$$

where $\overset{-}{z}$ and $\overset{-}{u}$ are the average value of time series and *u*. Then, the weight *w*of each series in this scale *s* is defined as follows:

(5) $$\begin{array}{r} w\left(s\right)=\frac{R\left(j\right)}{\overset{s}{\underset{j=1}{\sum }}R\left(j\right)} \end{array}$$

Finally, WMMSE is defined as follows:

(6) $$\begin{array}{r} WMMSE\left(s\right)=\overset{s}{\underset{i=1}{\sum }}\text{w}\left(i\right)*SampEn\left(i\right) \end{array}$$

where *SampEn*(*i*) is the value of the sample entropy.

## Results

### Comparison Between WMMSE and MSE

**Figure 2:**
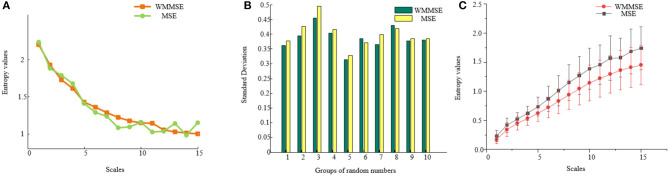
**(A)** Comparison between MSE and WMMSE. The x-axis represents scales, and the y-axis represents entropy values of traditional MSE and WMMSE. **(B)** The standard deviation of traditional MSE and WMMSE. The x-axis represents the group, and the y-axis represents the standard deviation of traditional MSE and WMMSE. **(C)** Comparison of EEG results between WMMSE and traditional MSE. Clinical data from MCI patients are used to compare the two algorithms: traditional MSE and WMMSE.

To compare the traditional MSE with the WMMSE, taking 1,000 random numbers, the difference between traditional MSE and WMMSE was analyzed (Figure 2A). Compared with traditional MSE, the fluctuation of WMMSE values is lower. The changes at different scales are clearer and more apparent, indicating that WMMSE can effectively overcome the information loss problem of traditional MSE and can more accurately analyze signals.

A total of 10 groups of 1,000 random numbers were selected to calculate the traditional MSE and WMMSE values. The variances of traditional MSE and WMMSE were calculated (Figure 2B). The variance of WMMSE is generally lower than that of the traditional MSE. Therefore, the dispersion degree of WMMSE is low, and the trend of WMMSE change in different scales is less apparent.

To further compare MSE and traditional WMMSE methods, the effect of WMMSE and traditional MSE was compared to analyze EEG signals of patients with MCI (Figure 2C). The fluctuation of WMMSE values is lower than that of the traditional MSE values. The trend of WMMSE values is evident and stable, which indicates that the WMMSE overcomes the information loss problem of traditional MSE. WMMSE is conducive for mining the hidden components of details in EEG signals and more accurately analyzes the EEG signals of patients with MCI.

### Weighted Multiple Multiscale Entropy Analyses

**Figure 3:**
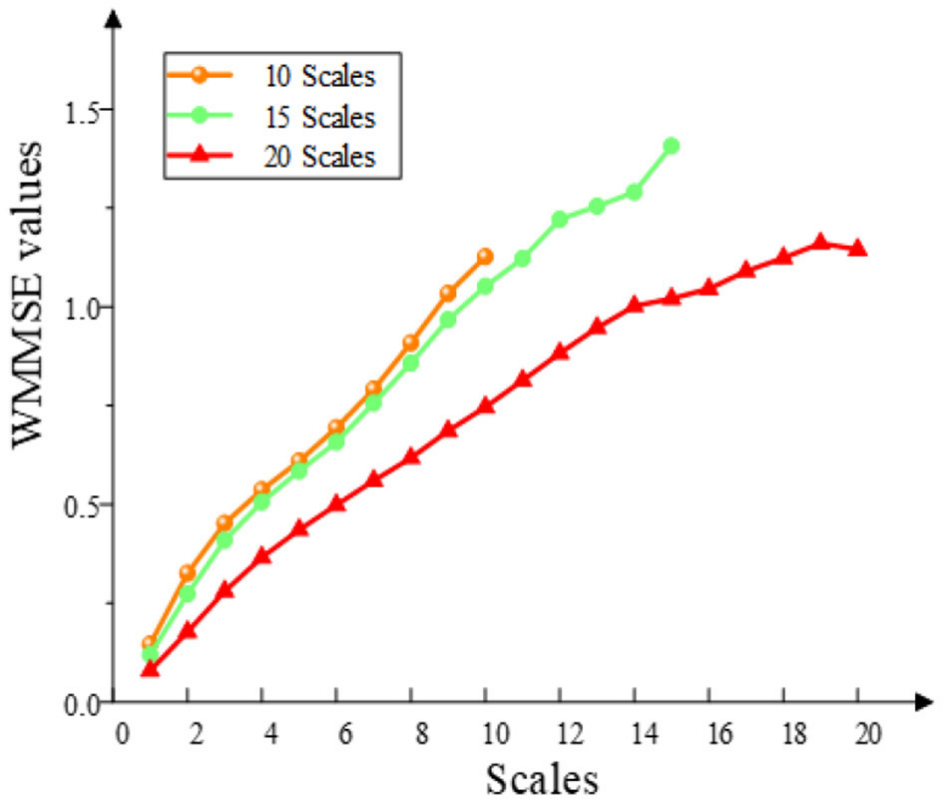
Selection of WMMSE scales. The WMMSE values are calculated when the multiscale parameters are 10, 15, and 20.

To calculate the WMMSE, three parameters are required, namely, embedding dimension (*m*), threshold (*r*), and decomposition scale (*s*). The parameter *m* is set to 2. The threshold *r* is based on the experience of calculating approximate entropy. *r* is generally set to 0.15 × std (*x*), where std (*x*) is the SD of the time series. When selecting scale parameters, experiments were carried out on 10, 15, and 20 scales (Figure 3). When 10 was chosen as the scale parameter, entropy values kept increasing. When 15 was selected as the scale parameter, the entropy values tend to be stable around 14 scales. When 20 was chosen as the scale parameter, the entropy values have a downward trend. The trend characteristics cannot be fully exploited since the scale is very low. However, the entropy distortion will be caused by the very high scale. When the scale was 15, the entropy fluctuated, but it was still rising. Therefore, setting the scale parameter to 15 was beneficial to the extraction of the MCI data information and avoided the complex calculation.

**Figure 4:**
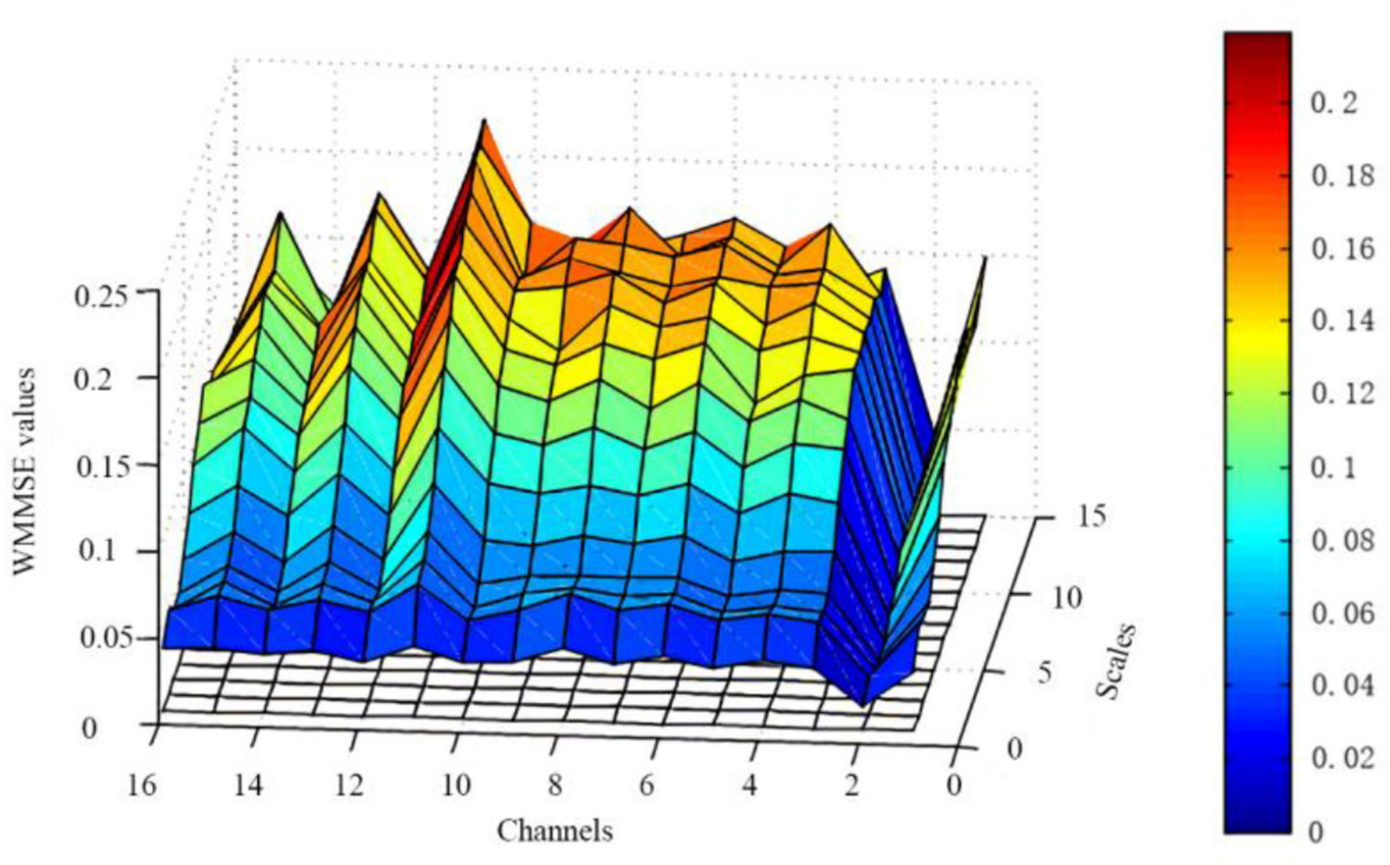
Changes of WMMSE values pre- and post- NFT. The x-axis represents 16 channels, the y-axis represents 15 scales, and the z-axis represents WMMSE values.

The embedding dimension (*m*), threshold (*r*), and decomposition scale (*s*) are based on the above results. The WMMSE pre- and post-NFT differences are analyzed for the EEG signals (i.e., 16 channels and 15 scales) of 39 patients with MCI (Figure 4). These results show that the post-NFT entropy values are improved compared with pre-NFT entropy values.

### Electroencephalogram Signal Analysis Based on Lempel-Ziv Complexity

**Figure 5:**
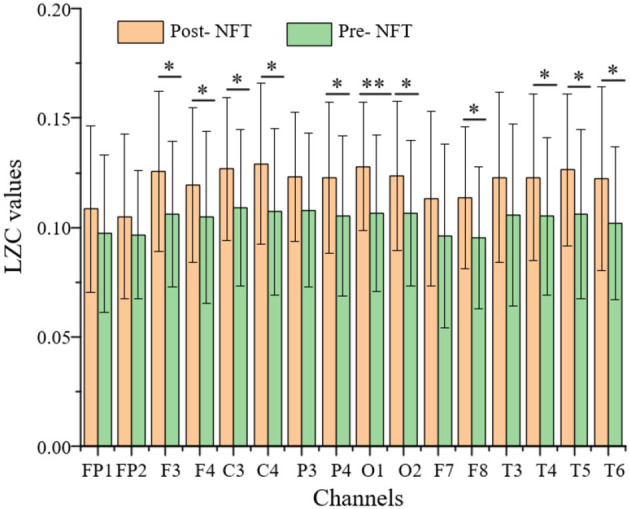
Comparison of LZC (pre- and post-NFT). The P-value of the paired t-test is expressed as follows: *P < 0.05 and **P < 0.01 are significant differences. The x-axis represents 16 channels, and the y-axis represents the LZC value.

Using the characteristics of Lempel-Ziv complexity (LZC), we found that the EEG signals of patients with AD have decreased complexity or increased regularity when compared with the healthy control groups (Simons and Abasolo, 2017; Steifer and Lewandowski, 2019). In this study, the EEG signals were obtained from 16 channels (i.e., 39 patients with MCI) in the frontal lobe, left and right temporal lobes, parietal lobe, and occipital lobe, both pre- and post-NFT were analyzed using LZC, and the LZC values post-NFT are higher than pre-NFT (Figure 5).

In Figure 5, there are significant differences (*P* < 0.05), pre- and post-NFT, in F3, F4, C3, C4, P4, O1, O2, F8, T4, T5, and T6 channels. Due to the symmetry of channel distribution, there is no significant difference in the symmetrical P3 and P4 channels, so they were not analyzed. Similarly, F7, F8, T3, and T4 were not analyzed. The LZC values of eight channels, namely, F3, F4, C3, C4, O1, O2, T5, and T6, were significantly higher post-NFT compared with pre-NFT.

Based on the LZC analysis, the WMMSE algorithm analyzes eight channels, namely, F3, F4, C3, C4, O1, O2, T5, and T6. The method of constructing weighted multiple sequences in each time scale overcomes the information loss problem of traditional MSE. Therefore, the results are more stable and better represent the complexity state or development direction of EEG signals at each time scale. Compared with the WMMSE, LZC can only evaluate its overall complexity and does not fully reflect the local multiscale details of the system. WMMSE is effective in mining the details of EEG signals and evaluates the effect of NFT more accurately.

**Figure 6:**
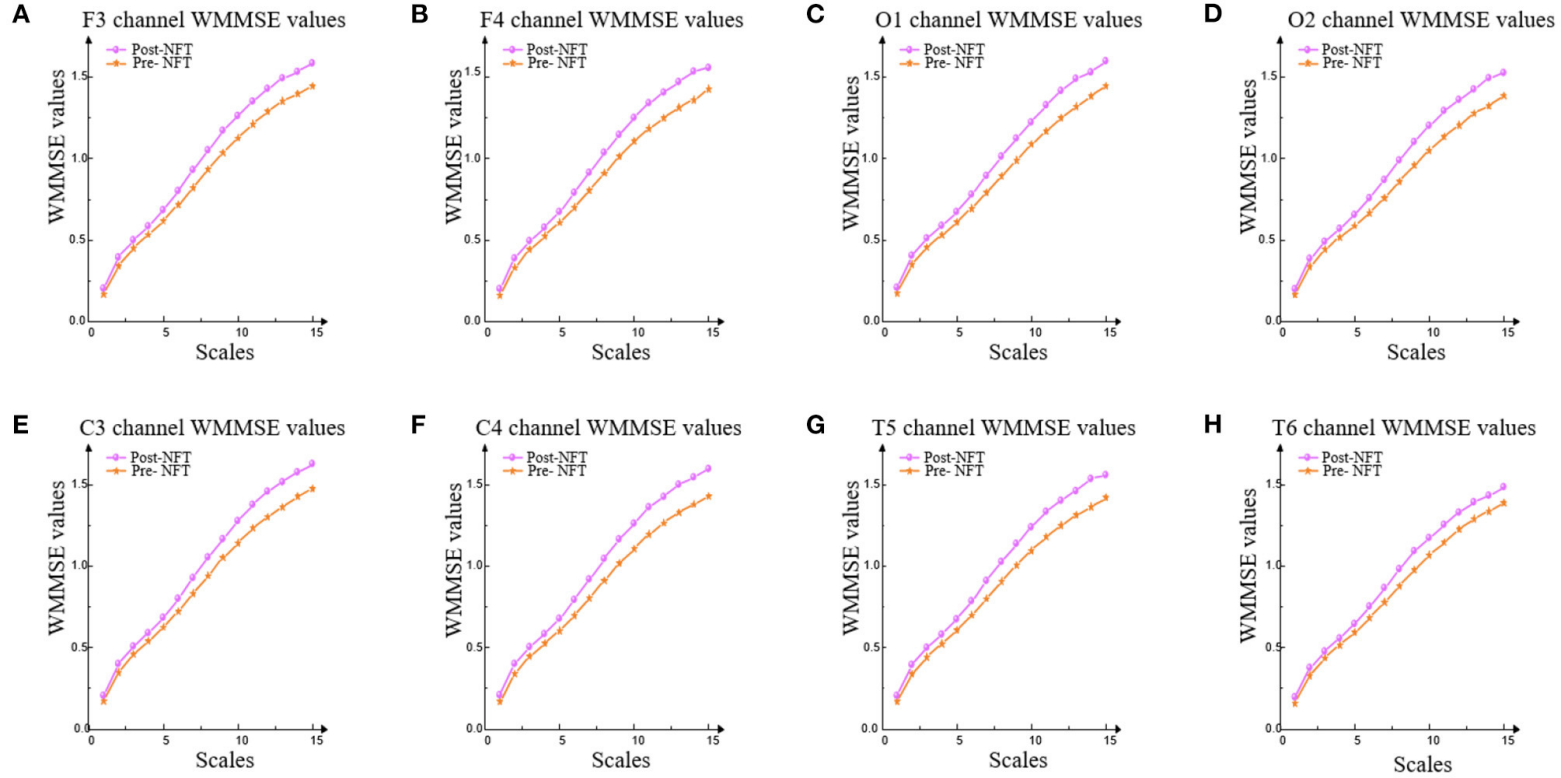
WMMSE values of eight channels. WMMSE values were used to analyze the values pre- and post-NFT in F3, F4, C3, C4, O1, O2, T5, and T6. **(A-H)** represent F3, F4, C3, C4, O1, O2, T5, and T6 channels, respectively.

The variation trend of WMMSE values pre- and post-NFT in F3, F4, C3, C4, O1, O2, T5, and T6 is shown in Figure 6.

In Figure 6, the trend of WMMSE values pre- and post-NFT is consistent. The results show that the WMMSE values of post-NFT are higher than pre-NFT on each scale in the eight channels (i.e., F3, F4, C3, C4, O1, O2, T5, and T6).

In Table 1, the results of the significance tests for the WMMSE values show that there are significant differences (*P* < 0.05) in F4, C3, C4, O1, and T5 channels pre- and post-NFT. Based on the LZC results, F3, O2, and T6 channels are excluded. The WMMSE fully reflects the ability to mine the hidden details of EEG signals, which is more accurate than the result of LZC.

**Table 1:** Significance test results of WMMSE pre- and post-NFT.

**Channel**	**Significant (*P*-value)**	**Channel**	**Significant (*P*-value)**
F3	0.067	F4	0.028
C3	0.017	C4	0.041
O1	0.011	O2	0.083
T5	0.049	T6	0.255

**Figure 7:**
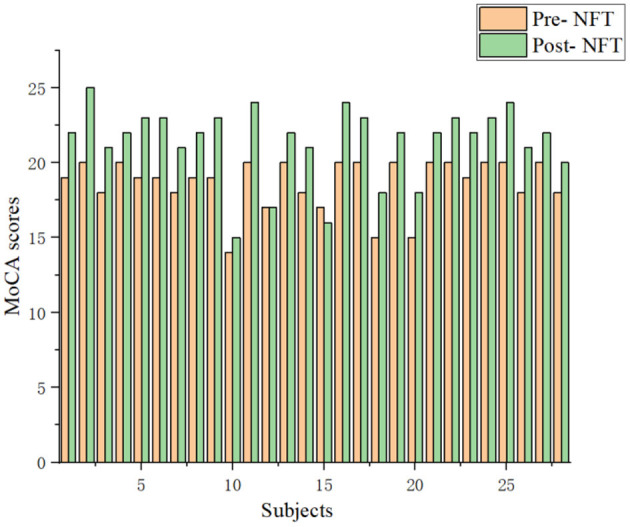
Comparison of the Montreal Cognitive Assessment (MoCA) scores with the patients with MCI between pre- and post-NFT.

The Montreal Cognitive Assessment (MoCA) (Rosca et al., 2020) score is an essential clinical index for MCI. MoCA has high sensitivity and specificity in the diagnosis of patients with MCI (Thomas et al., 2018). Twenty eight patients with MCI were randomly selected from 39 patients. Post-NFT scores are higher than pre-NFT scores in the vast majority of 28 patients with MCI (Figure 7). Therefore, the NFT can improve brain function. However, 1 patient with MCI does not change pre- and post-NFT. The MoCA score of 1 patient with MCI was lower by 1. Thus, the long-term NFT is needed to regulate the brain functions of patients with MCI.

**Figure 8:**
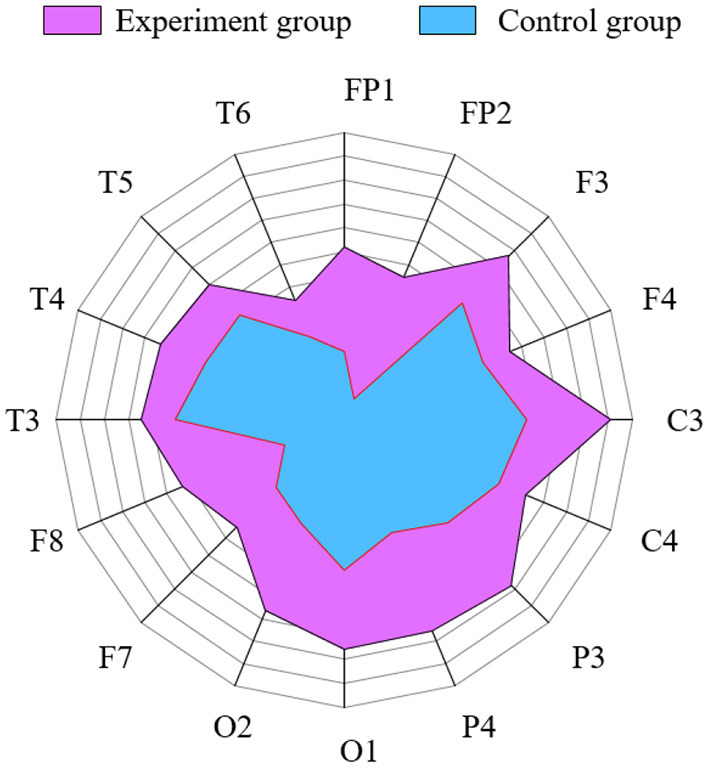
Comparison of WMMSE values between the experimental and control group in each channel. Radius represents WMMSE values.

The control group and the experiment group treated with NFT were analyzed using the WMMSE measure (Figure 8). The circle is divided into 16 parts representing 16 channels. The radius of the circle represents the WMMSE value of each channel. Figure 8 demonstrates that the experimental group area is significantly larger than that of the control group, and the above results have significant differences (*P* < 0.05) using the independent sample *t*-test method. Therefore, the WMMSE values of the experimental group are greater than that of the control group in every channel.

## Discussion

This study presented an NFT method to interfere with the brain function of patients with MCI. Meanwhile, to analyze the NFT effect, this study also proposed a new evaluation algorithm (i.e., WMMSE). The results indicated that NFT and WMMSE may improve the cognitive function of patients with MCI. This study extends the relative studies on the MCI brain function.

In many studies, MCI has become the focus of early diagnosis of AD. The previous study has been found that a new method called a correlation-based label consistent K-SVD can diagnose patients with MCI (Kashefpoor et al., 2019). Brain functional connectivity has been analyzed the changes from MCI to AD based on resting and cognitive task conditions (Surya and Puthankattil, 2020). To diagnose patients with MCI, the permutation entropy neuromarker was proposed based on EEG signals (Eker et al., 2021). In this study, the brain function of patients with MCI was intervened and evaluated.

In recent years, many studies have tried to intervene in the dysfunctional regulation of cortical by neurofeedback (Vernon et al., 2003). The previous study has found that NFT may improve the brain function of patients with MCI based on EEG signals (Marlats et al., 2020). Some studies have also analyzed neurofeedback that could improve memory based on EEG signals with patients with MCI (Lavy et al., 2019). In this study, the NFT was used to improve the brain function of patients with MCI. The results showed that the NFT can improve the complexity of EEG signals to a certain extent. With this in mind, the NFT could be used to intervene the cognitive decline. The result was similar to recent findings in which NFT as a technique can improve the cognitive decline earlier (Hanslmayr et al., 2005; Becerra et al., 2012).

This study has supplied and discussed the progression and difference from the previous study (Li X. et al., 2019). First, in the previous study, the non-linear method WMMSE was used to analyze the non-linear dynamics of ASD based on EEG signals. The previous study showed that WMMSE can analyze the non-linear dynamics and extract more accurate features from the EEG signals of ASD. However, in this study, we investigated that whether the WMMSE can analyze the non-linear dynamics of patients with MCI based on the EEG signals. Second, this study further analyzed the parameter based on our previous study on the WMMSE method. More specifically, the weight parameter, multiscale parameter, and parameter (*l*) of the WMMSE method were analyzed in this study. For the weight parameter, when the correlation coefficient was calculated, the data length ({*z*^(*s*)^}) of the coarse-graining was equal to the data length ({*u*^(*s*)^}) of the interval value. For the multiscale parameter, in our previous study on ASD, when the scale was 15, the entropy data were relatively stable. When the scale was 20, our previous studies did not make a detailed analysis. However, in this study, we analyzed the influence of different scales on entropy. Based on the MCI clinical data, when the scale was 15, the entropy fluctuated, but it was still rising. When the scale was 20, the entropy had a downward trend. Therefore, setting the scale parameter to 15 was beneficial to the extraction of MCI data information and avoided complex calculations. For the parameter (*l*), it was defined as the largest integer no more than itself for solving the question on different EEG signals.

In this study, the WMMSE and the LZC were used to analyze the effect of patients with MCI on improving brain function. The results have shown that the LZC of the EEG signal (i.e., post-NFT) was higher than that of pre-NFT in F3, F4, C3, C4, P4, O1, O2, F8, T4, T5, and T6 channels, and there were significant differences in six channels. The WMMSE algorithm was used to analyze the LZC results further. The WMMSE post-NFT was higher than pre-NFT in the channels F4, C3, C4, O1, and T5, and there were significant differences. Compared with the LZC, the WMMSE excluded three channels (i.e., F3, O2, and T6). The results showed that the WMMSE extracted the hidden components of EEG signals and was more accurate than the LZC in the evaluation of NFT. Therefore, WMMSE can accurately and effectively evaluate the improvement effect of NFT on the brain function of patients with MCI.

In addition, the neuropsychological scale was used to verify the effect of NFT. The MoCA scores of 28 patients with MCI, pre- and post-NFT, were analyzed. MoCA scores of 26 patients with MCI increased, but 1 patient with MCI did not change pre- and post-NFT, and 1 had a MoCA score that was lower by 1. This result also further proves that NFT can improve brain cognitive functional recovery of MCI from a clinical perspective. In future studies, the efficiency of the long-term NFT on cognitive ability will be analyzed.

## Conclusions

In this study, we proposed the WMMSE method to explore the characteristics of the EEG signal of patients with MCI after NFT. Our results showed that the WMMSE method performed better compared with the traditional MSE method and LZC, and it is found that the WMMSE can more accurately evaluate the effect of NFT on brain function. Further analysis of the clinical data showed that the WMMSE post-NFT is higher than pre-NFT in channels F4, C3, C4, O1, and T5, and there were significant differences (*P* < 0.05). Meanwhile, MoCA scores pre- and post-NFT were analyzed, and the MoCA scores of most patients with MCI post-NFT were higher than pre-NFT. Both the simulation and clinical data expounded on the effectiveness of the proposed WMMSE measure to estimate the efficiency of NFT. Therefore, WMMSE may be a new analysis method to measure the effect of NFT accurately and may aid in furthering the MCI research.

## Data Availability Statement

The original contributions presented in the study are included in the article/supplementary material, further inquiries can be directed to the corresponding author.

## Ethics Statement

The studies involving human participants were reviewed and approved by the ethical review board of Yanshan University. The patients/participants provided their written informed consent to participate in this study. Written informed consent was obtained from the individual(s) for the publication of any potentially identifiable images or data included in this article.

## Author Contributions

RS: writing-original draft preparation, methodology, and data curation. XL: writing-reviewing and editing and supervision. YL: data curation. WC: medical support. PX: supervision. YH: medical support. All authors contributed to the article and approved the submitted version.

## Conflict of Interest

The authors declare that the research was conducted in the absence of any commercial or financial relationships that could be construed as a potential conflict of interest.

## Publisher's Note

All claims expressed in this article are solely those of the authors and do not necessarily represent those of their affiliated organizations, or those of the publisher, the editors and the reviewers. Any product that may be evaluated in this article, or claim that may be made by its manufacturer, is not guaranteed or endorsed by the publisher.
